# The chronic leukocyte and inflammatory cytokine responses of older adults to resistance training in normobaric hypoxia; a randomized controlled trial

**DOI:** 10.1186/s13102-024-00890-w

**Published:** 2024-05-02

**Authors:** Giselle Larissa Allsopp, Alex Bernard Addinsall, Garth Stephenson, Faiza Basheer, Paul Adrian Della Gatta, Samantha May Hoffmann, Aaron Paul Russell, Craig Robert Wright

**Affiliations:** 1 Institute for Physical Activity and Nutrition, School of Exercise and Nutrition Sciences, Deakin University, Victoria, Australia; 2 Department of Physiology and Pharmacology, Karolinska Insitutet, Stockholm, 171 77 Sweden; 3 School of Medicine, Deakin University, Geelong, Victoria Australia; 4 Institute for Mental and Physical Health and Clinical Translation (IMPACT), Deakin University, Geelong, Victoria Australia; 5 Centre for Sport Research (CSR), School of Exercise and Nutrition Sciences, Deakin University, Victoria, Australia

**Keywords:** Simulated altitude, White blood cells, Aging, Strength training, Inflammation

## Abstract

**Trial design:**

Older adults experience chronic dysregulation of leukocytes and inflammatory cytokines, both at rest and in response to resistance training. Systemic hypoxia modulates leukocytes and cytokines, therefore this study characterized the effects of normobaric hypoxia on the leukocyte and cytokine responses of older adults to resistance training.

**Methods:**

20 adults aged 60–70 years performed eight weeks of moderate-intensity resistance training in either normoxia or normobaric hypoxia (14.4% O_2_), consisting of two lower body and two upper body exercises. Venous blood was drawn before and after the training intervention and flow cytometry was used to quantify resting neutrophils, lymphocytes, monocytes, eosinophils and basophils, in addition to the subsets of lymphocytes (T, B and natural killer (NK) cells). Inflammatory cytokines were also quantified; interleukin 1 beta (IL-1β), IL-4, IL-6, IL-8, IL-10 and tumor necrosis factor alpha (TNF-α). Acute changes in leukocytes and cytokines were also measured in the 24 h following the last training session.

**Results:**

After the intervention there was a greater concentration of resting white blood cells (*p* = 0.03; 20.3% higher) T cells (*p* = 0.008; 25.4% higher), B cells (*p* = 0.004; 32.6% higher), NK cells (*p* = 0.012; 43.9% higher) and eosinophils (*p* = 0.025; 30.8% higher) in hypoxia compared to normoxia, though the cytokines were unchanged. No acute effect of hypoxia was detected in the 24 h following the last training session for any leukocyte population or inflammatory cytokine (*p* < 0.05).

**Conclusions:**

Hypoxic training caused higher concentrations of resting lymphocytes and eosinophils, when compared to normoxic training. Hypoxia may have an additional beneficial effect on the immunological status of older adults.

**Trial registration:**

Australian New Zealand Clinical Trials Registry (ANZCTR). Trial number: ACTRN12623001046695. Registered 27/9/2023. Retrospectively registered. All protocols adhere to the COSORT guidelines.

**Supplementary Information:**

The online version contains supplementary material available at 10.1186/s13102-024-00890-w.

## Background

Aging is associated with a gradual decline in the number and function of circulating leukocytes (white blood cells) that has been coined ‘immunosenescence’ [1]. The consequences of immunosenescence include an increased incidence and severity of infections such as influenza [2] and sepsis [3]. The mortality rate from simple infections such as urinary-tract infection is ten-fold higher in older adults when compared to younger adults [4]. The causes of immunosenescence are not entirely clear, though they include thymic involution, persistent exposure to pathogens across the lifespan and altered hormone concentrations including cortisol, adrenaline and growth hormone [1].

The hallmarks of immunosenescence include reduced functional capacity of neutrophils [5] and decreased eosinophil degranulation [6]. The resting number of classical monocytes also declines with age [7], likely reducing the phagocytic and migratory capacity of these cells [8]. Of particular interest in older adults is lymphocyte senescence, where a decline in lymphocyte number and function increases their susceptibility to illness [9]. Of the three lymphocyte subpopulations, the quantity of natural killer (NK) cells typically increases with age [10], whilst their cytolytic capacity is potentially blunted [11]. Although the number of circulating T and B lymphocytes is largely unchanged in older adults at rest, there is an increased proportion of highly differentiated memory T and B lymphocytes that are less responsive to antigenic stimulation [12, 13]. The T lymphocyte subpopulations also show signs of senescence where the normal 2:1 ratio of CD4^+^ helper T cells to CD8^+^ cytotoxic T cells is reduced to less than 1:1 [14]. CD4:CD8 T cell inversion is typically caused by an increased number of late-stage differentiated CD8^+^ T cells that possess a limited proliferative and cytolytic capacity [15]. Collectively, these changes reduce the capacity of older adults to respond to antigenic stimuli and can profoundly impact their health, immunity and longevity [16].

Ageing is also associated with cytokine dysregulation termed ‘inflammageing’, where circulating pro-inflammatory cytokines such as tumor necrosis factor alpha (TNFα) are chronically elevated [17] and anti-inflammatory cytokines are reduced [18]. These high concentrations of systemic TNFα and interleukin-6 (IL-6) in adults aged over 65 are associated with muscle weakness [19] and frailty [20]. Inflammation and leukocyte dysfunction in the ageing population are therefore clearly linked to poor clinical outcomes and must be targeted to promote healthy ageing.

Growing evidence shows that chronic resistance training alters the milieu of immune cells in circulation, though these responses are blunted in older adults. For example, the acute NK cell response following intense resistance exercise was blunted in older adults compared to young adults [21]. Interestingly, this response was partially restored after eight-weeks of high-intensity resistance training [21]. Older adults also show a shorter duration of increase in monocytes and lymphocytes following acute resistance exercise that is irreversible with 21 weeks of resistance training [22]. A coordinated lymphocyte response is vital to effectively eliminate pathogens, therefore blunted lymphocyte responses may reduce resistance to infection in older adults, when compared to young adults. Furthermore, resistance training does not consistently improve red blood cell or haemoglobin concentrations in older adults [23], despite its potential effectiveness in younger populations [24]. Although reduced circulating C-reactive protein (CRP) is consistently observed following resistance training in older adults, TNFα and IL-6 are not consistently reduced [25]. Resistance training is only partially effective at slowing the progression of immunosenescence and inflammageing. Therefore, novel exercise interventions are needed to better treat these conditions.

We previously showed that hypoxia does not amplify the lean mass, muscle strength, muscular endurance or aerobic endurance adaptations of older adults to eight-weeks of resistance training compared to the same training in normoxia [26]. We also showed that resting and post-exercise concentrations of insulin, testosterone and cortisol are unresponsive to eight-weeks of resistance training in hypoxia, and the post-exercise growth hormone response was blunted by hypoxia following the last training session of the intervention [27]. Despite these results, the literature consistently shows that acute hypoxic treatment modulates immunological variables. For example, exposure to hypoxia for 20 min increased the number and function of circulating NK cells and increased monocyte concentrations in healthy individuals [28]. Hypoxic resistance training was originally utilized with the aim to increase training adaptations in young adults [29], though more recent research recruited older adults. Our research group previously characterized the acute immunological responses of untrained older adults to resistance exercise in normobaric hypoxia [30]. Here, we showed that a single bout of resistance exercise in normobaric hypoxia increased the number of circulating lymphocytes in older adults up to 24 h post-exercise, to a greater extent than the same exercise in normoxia. However, it remains unknown if prolonged (eight weeks) of resistance exercise in hypoxia alters resting levels of leukocytes in older adults or the post-exercise response.

To follow up our findings on the acute immunological responses to a single bout of hypoxic resistance exercise, this study used previously collected samples to characterize the chronic leukocyte and inflammatory cytokine responses of older adults to eight-weeks of resistance training in hypoxia. We hypothesized that resistance training in hypoxia for eight weeks would cause a greater increase in the resting concentration of leukocytes and anti-inflammatory cytokines, and a greater decrease in pro-inflammatory cytokines compared to the same training in normoxia. We also hypothesized that after the eight-week training intervention, the hypoxic group would show greater acute leukocyte and cytokine responses to the resistance training protocol, compared to the same exercise in normoxia.

## Methods

### Study design

As part of a larger study [27, 30, 31], a single-blinded randomized intervention investigated the effects of resistance training in normobaric hypoxia on the primary outcome; chronic leukocytes (specifically lymphocytes) and the secondary outcome; inflammatory cytokines in older adults. The experimental methods used in this study are previously described and the participant characteristics were previously reported [26]. In brief, healthy adults aged 60–75 years were invited into eight weeks of supervised resistance training in either normobaric hypoxia or normoxia. The final recruitment included twenty healthy, recreationally active, non-resistance trained males and females aged 60–70 years (12 males and 8 females; Table 1). Participant characteristics were measured during an initial visit to the research center; age, body mass, body mass index (BMI), V̇O_2_ peak and blood pressure. The senior researcher randomly allocated participants to the hypoxic or normoxic group in blocks of 4 participants, based on the time of their enrolment to the trial (1:1 allocation ratio). Participants had no diagnosed history of cardiovascular or respiratory disease, were not taking regular medication and did not report any illness over the period of the study. All trials were performed at the School of Exercise and Nutrition Sciences, Deakin University, Geelong between 2018 and 2019.

A power analysis was performed to confirm that this study was adequately powered to detect changes in our primary outcome measure (lymphocytes). A priori repeated measures analysis of variance (ANOVA) was performed in G*Power, within-between factors (the F test; Version 3.1.9.4; Universität Kiel, Germany). Resting lymphocyte concentrations were selected following a resistance training intervention with two experimental conditions; high fat and high carbohydrate diet [32]. Cohens d was calculated and then transformed into effect size f. The parameters included alpha error probability (0.05), power (1-β error probability; 0.80), groups (two), number of measurements (two; pre and post intervention), standard correlation among repeated measures (0.5), non-sphericity correction (1.0). Therefore, to detect an effect size (f) of 0.65 using a two-way repeated measures ANOVA for mean lymphocyte count, a minimum of 8 participants were required. To account for potential drop out, 10 participants were recruited per group.

**Table 1:** Participant characteristics (mean ± SD). *n* = 6 males and *n* = 4 females in each group. Previously reported as part of a larger study [26]

	Normoxia (*n* = 10)	Hypoxia (*n* = 10)
Age (years)	64.0 ± 0.8	65.9 ± 1.1
Body mass (kg)	71.9 ± 4.3	70.7 ± 4.4
BMI (kg/m^2^)	23.9 ± 0.8	24.9 ± 1.1
V̇O_2_peak (ml/kg/min)	30.3 ± 1.9	28.2 ± 3.4
Systolic blood pressure (mmHg)	130.2 ± 5.2	133.6 ± 7.3
Diastolic blood pressure (mmHg)	83.6 ± 2.7	83.0 ± 3.3

V̇O_2_peak was measured on a cycle ergometer (Lode Excalibur Sport, Netherlands) using an incremental stepwise protocol that was previously validated for older adults. Participants wore a heart rate monitor (Polar T34, Polar Electro Oy, Finland) and a respiratory mask during the test. After a 2-minute warm up at 50 W, the load was increased by 5 W every 30s (females) and 5 W every 20s (males) until a plateau in oxygen consumption was reached, or volitional fatigue.

Normobaric hypoxia was achieved in an environmental tent using a normobaric hypoxic generator (Pulford Systems, Australia), as previously described [26]. Briefly, ambient air was enriched with N_2_ to reduce the O_2_ in the tent to 14.4%, the equivalent altitude of approximately 3,000 m above sea level [33]. To blind participants from their group allocation, those in the normoxic group also completed their sessions in the environmental tent with the generator running and set to 20.93% O_2_.

Participants performed two resistance training sessions per week for eight weeks. Sessions consisted of four whole-body exercises performed at 70% of each participant’s predicted 1-repetition maximum (RM; in the following order); leg extension, pectoral fly, standing row and squat. Participants performed 4 sets of 10 repetitions for each exercise with a 1-minute rest between sets and a 2-minute rest between exercises. The methods used to measure maximum strength are previously described, where 1RM was estimated from 5RM using the following formula; 1RM = 5RM kg lifted/(1.0278–0.0278 × the number of repetitions) [26]. Participants attended 100% of their training sessions and there was no attrition during the study period. The total load performed by each group over the eight-week period was not significantly different (total load was assessed by calculating the total number of sets, reps and weight lifted over each of the sessions in the eight-week training period; *p* > 0.05). At the end of the intervention, participants were asked to guess which group they were allocated to. Participants were not consistently able to guess their group allocation, with 18/20 participants guessing that they were allocated to hypoxic training.

### Blood sampling

5mL of venous blood was sampled at the start (week 0) and the end of the eight-week training intervention (week 8; immediately before and in the hours following the final training session) from an antecubital vein and was transferred into an EDTA vacutainer (BD, USA). Blood samples were also collected following the last training session in week eight, to characterize the acute leukocyte and inflammatory cytokine responses to hypoxic resistance exercise following a period of training (pre, post 0,15,30,60,120,180 min and 24 h).

### Hematology

A full blood count (FBC) was performed on the EDTA-treated whole blood samples using a hematology analyzer (DhX500, Beckman Coulter, Australia) to quantify the concentration of total leukocytes and their 5 major subsets; neutrophils, lymphocytes, monocytes, eosinophils, and basophils. Blood samples were analyzed at rest before and after the eight-week training intervention. Blood was also analyzed at 0,15,30,60,120,180 min and 24 h after the last training session of the intervention. The FBC also quantified RBCs, red cell distribution width (RDW), hematocrit, hemoglobin, mean corpuscular volume (MCV), mean corpuscular hemoglobin (MCH), MCH concentration (MCHC), platelets and mean platelet volume (MPV). The %CV for leukocyte, lymphocyte, monocyte, neutrophil, eosinophil, basophil and platelet measurements was 1.2, 3.7, 4.0, 1.8, 15.1, 10.5 and 8.2% respectively.

### Flow cytometry

Determination of the leukocyte subpopulations was achieved using flow cytometry with multi-color analysis (BD FACSCanto II, BD Biosciences, Australia; FACSDIVA v9.0 software). Blood samples were analyzed at rest before and after the eight-week training intervention. Blood was also analyzed at 0, 180 min and 24 h after the last training session of the intervention. The preparation of samples was previously described by our research group [31]. The three lymphocyte subpopulations (NK cells, T cells and B cells) were quantified and the two subsets of T cells were also quantified; CD4^+^ (helper) T cells and CD8^+^ (cytotoxic) T cells. The expression of cluster of differentiation 45RA (CD45RA) on CD4^+^ and CD8^+^ T cells was used as a basic indicator of cell senescence, where naïve T cells are typically CD45RA^+^ and senescent T cells are CD45RA^−^ [34].

Total monocytes and the monocyte subpopulations (classical, non-classical and intermediate) were also identified. Due to a low number of monocyte cells in each sample, it was not possible to identify the three monocyte subpopulations; For this reason, monocytes were identified either as classical (CD14^+^ CD16^−^) or non-classical (CD14^+^ CD16^+^), meaning that the small number of intermediate cells were likely included in the non-classical population.

### Cytokine analysis

Venous plasma samples were analyzed in duplicate using a high sensitivity human cytokine Milliplex T cell panel (MPHSTCMAG28SK06; Merck Millipore, Abacus Dx, Australia) to quantify the protein concentration of inflammatory cytokines; IL-1β, IL-4, IL-6, IL-8, IL-10 and TNFα. Blood samples were analyzed at rest before and after the eight-week training intervention. Blood was also analyzed at 0, 180 min and 24 h after the last training session of the intervention. The average intra-assay %CV for IL-1β, IL-4, IL-6, IL-8, IL-10 and TNFα was 3.4, 5.5, 7.5, 8.3, 8.4 and 6.3%, and the average inter-assay %CV was 8.7, 6.5, 7.4, 6.2, 7.7 and 8.2%, respectively. The minimum detection limit for IL-1β, IL-4, IL-6, IL-8, IL-10, and TNFα was 0.14, 1.12, 0.11, 0.13, 0.56 and 0.16 pg/mL, respectively. Given that plasma can shift between the blood and the extracellular space during exercise, plasma volume shift was accounted for in all blood analyses using the Dill and Costill method [35].

### Statistical analysis

Statistical analysis was completed using SPSS (IBM SPSS 26, Chicago, IL) and graphed using GraphPad software (Prism 8.00, California USA). All data were assessed for normal distribution using a Shapiro-Wilk test, and any variables that failed the Shapiro-Wilk test underwent Log_10_ transformation prior to analysis. The chronic leukocyte, hematology and cytokine adaptations to the eight-week intervention were analyzed using an ANCOVA with group as the independent variable (two levels; normoxia, hypoxia) for the main effect of time (two levels; pre-training, post-training) and the time × group interaction. Sex and pre-training values were included as covariates. Data are presented as adjusted mean ± SEM, with delta change (post value - pre value) and 95% confidence intervals (95% CI). Significance was set at *p* < 0.05.

To examine magnitude-based inferences in resting leukocytes, hematological parameters and inflammatory cytokines, effect size was calculated on the mean difference for each variable (the difference between the delta change for normoxia and the delta change for hypoxia). To avoid the positive bias associated with Cohens *d* in small sample sizes, Hedges *g* statistic was computed with a bias correction using an Excel template. The precision of mean differences was calculated with 95% CI, to express the range of uncertainty of the interval containing the true parameter value. Qualitative descriptors of standardized (Cohen’s *d*) effect sizes were assessed using these criteria: trivial < 0.2, small 0.2–0.49, moderate 0.5–0.79, and large ≥ 0.8. Effects with 95% CIs overlapping the thresholds for small positive and small negative effects (i.e., exceeding 0.2 of the 95% CIs on both sides of zero) were defined as “unclear.” A “clear” effect size was defined as the mean of the 95% CI not exceeding 0.2 on the other size of zero.

The acute leukocyte and cytokine responses to the final session of the eight-week intervention were analyzed using an ANCOVA with group as the independent variable (two levels; normoxia, hypoxia) for the main effect of time and the time × group interaction. For the leukocyte populations, there were eight levels of time (pre-exercise, post 0,15,30,60,120 and 180 min, 24 h post-exercise) and for the flow cytometry and cytokine variables, there were four levels of time (pre-exercise, post 0, 3 h, 24 h). If a time × group interaction was present, a Bonferroni post-hoc test was used to evaluate the effect of hypoxia on individual time points (6 possible pairwise time contrasts). Sex and pre-training values were included as covariates. Changes to the leukocyte populations over time (irrespective of group allocation) were analyzed using pairwise comparisons of the pooled data based on the estimated marginal means with Bonferroni adjustment. Data are presented as adjusted mean ± SD.

## Results

### Chronic leukocyte responses to the training intervention

There was a significant time × group interaction for total white blood cells (leukocytes; *p* = 0.030), leukocytes (*p* = 0.013), T lymphocytes (*p* = 0.012), CD8^+^ cytotoxic T cells (*p* = 0.003), NK cells (*p* = 0.004) and eosinophils (*p* = 0.025), where these populations were significantly higher after 8 weeks of resistance training in hypoxia compared to normoxia, and displayed a large effect (Table 2).

After the intervention, resting B lymphocytes were higher in the hypoxic group compared to normoxia (time × group interaction; *p* = 0.008) however, the effect size analysis was unclear.

No significant interactions were observed for basophils, neutrophils, total monocytes or the monocyte subsets of CD14^+^ CD16^−^ and CD14^+^ CD16^+^ cells, CD4^+^ T helper cells nor the expression of CD45RA on CD4^+^ T helper cells or CD8^+^ T cytotoxic cells. However, there was a significant main time effect for neutrophils, where neutrophils decreased following 8 weeks of resistance training, irrespective of the hypoxia exposure (*p* = 0.013).

**Table 2:** Chronic WBC responses to eight weeks of resistance training in hypoxia; Total WBCs, neutrophils, lymphocyte subsets (and their CD45RA expression), monocytes (and CD14/CD16 expression) eosinophils and basophils at pre- and post-training. Values are adjusted mean ± SE. *N* = 10. Adjusted values are reported from the ANCOVA output, using baseline and sex as covariates. The p value reported is the time × group interaction

	Pre training	Normoxia		Hypoxia		Between group differences	
	Adjusted Mean ± SE	Post training Mean ± SE	Δ Change [95%C.I]	Post training Mean ± SE	Δ Change [95%C.I]	*P* value	Hedges G [95% CI]
White blood cells (×10^9^/L)	4.98 ± 0.00	4.17 ± 0.27	−0.81 [−1.44, 0.18]	5.11 ± 0.23	0.13 [−0.43, 0.69]	0.030	1.15 ± [0.15, 2.15]
Neutrophils (×10^9^/L)	2.71 ± 0.00	2.35 ± 0.21	−0.36 [−0.86, 0.14]	2.56 ± 0.19	−0.16 [−0.61, 0.29]	0.520	0.33 [−0.61, 1.27]
Lymphocytes(×10^9^/L)	1.66 ± 0.00	1.38 ± 0.09	−0.28 [−0.48, −0.08]	1.83 ± 0.08	0.17 [−0.01, 0.35]	0.001	1.90 [0.78, 3.02]
CD19^+^ B lymphocytes (×10^9^/L)	0.20 ± 0.00	0.18 ± 0.02	−0.02 [−0.07, 0.03]	0.25 ± 0.02	0.05 [0.00, 1.00]	0.008	1.38 [0.35, 2.41]
CD56^+^ Natural killer cells (×10^9^/L)	0.25 ± 0.00	0.16 ± 0.02	−0.09 [−0.14, −0.04]	0.25 ± 0.02	0.00 [0.23, 0.27]	0.004	1.87 [0.72, 3.02]
CD3^+^ T lymphocytes(×10^9^/L)	1.19 ± 0.00	1.03 ± 0.08	−0.16 [−0.34, 0.02]	1.33 ± 0.06	0.14 [−0.01, 0.29]	0.012	1.54 [1.1, 0.44]
CD4^+^ T helper cells (×10^9^/L)	0.83 ± 0.00	0.75 ± 0.07	−0.08 [−0.39, 0.23]	0.92 ± 0.05	0.09 [0.04, 0.22]	0.090	0.97 [−0.05, 1.99]
CD45RA^+^ (% of CD4^+^ cells)	47.8 ± 0.0	49.6 ± 1.8	1.8 [−3.0, 6.6]	47.0 ± 1.6	−0.9 [−4.6, 2.8]	0.298	0.55 [−0.4, 1.5]
CD45RA^−^ (% of CD4^+^ cells)	52.2 ± 0.0	50.4 ± 1.8	−1.8 ± [−6.6, 3]	53.0 ± 1.6	0.9 [−2.8, 4.6]	0.298	0.55 [−0.4. 1.5]
CD8^+^ T cytotoxic cells (×10^9^/L)	0.36 ± 0.00	0.28 ± 0.03	−0.08 [−0.14, −0.02]	0.40 ± 0.02	0.04 [−0.01, 0.09]	0.003	1.93 [0.77, 3.09]
CD45RA^+^ (% of CD8^+^ cells)	67.5 ± 0.0	68.9 ± 1.7	1.4 [−2.8, 5.6]	67.7 ± 1.6	0.2 [−3.4, 3.8]	0.625	0.24 [−0.66, 1.14]
CD45RA^−^ (% of CD8^+^ cells)	32.5 ± 0.0	31.2 ± 1.7	−1.4 [−5.6, 2.8]	32.3 ± 1.6	−0.2 [−0.362, 3.58]	0.625	0.24 [−0.66, 1.14]
CD4^+^:CD8^+^ ratio	2.84 ± 0.00	3.14 ± 0.19	0.30 [−0.14, 0.74]	2.74 ± 0.16	−0.10 [−0.47, 0.27]	0.149	0.58 [−0.41, 1.57]
Monocytes (×10^9^/L)	0.47 ± 0.00	0.43 ± 0.02	−0.04 [−0.09, 0.01]	0.47 ± 0.02	0.00 [−0.01, 0.01]	0.138	0.69 [−0.4, 1.78]
CD14^+^ CD16^+^ (% of monocytes)	16.7 ± 0.0	15.5 ± 1.8	−1.2 [−5.3, 2.9]	16.3 ± 1.7	−0.4 [−4.3, 3.5]	0.740	0.17 [−0.73, 1.07]
CD14^+^ CD16^−^ (% of monocytes)	83.3 ± 0.0	84.5 ± 1.8	1.2 [−2.9, 5.3]	83.7 ± 1.7	0.4 [−3.5, 4.3]	0.740	0.17 [−0.73, 1.07]
Eosinophils (×10^9^/L)	0.13 ± 0.00	0.11 ± 0.02	−0.02 [−0.06, 0.02]	0.15 ± 0.01	0.02 ± [−0.01, 0.05]	0.025	1.08 [0.09, 2.07]
Basophils (×10^9^/L)	0.01 ± 0.00	0.01 ± 0.00	0.00 [−0.01, 0.01]	0.02 ± 0.00	0.00 [−0.01, 0.01]	0.310	0.09 [−0.84, 1.02]
Neutrophil/Lymphocyte ratio	1.76 ± 0.00	1.79 ± 0.17	0.03 [−0.36, 0.42]	1.45 ± 0.15	−0.31 [−0.66, 0.04]	0.095	0.91 [−0.07, 1.89]
Lymphocyte/monocyte ratio	3.69 ± 0.00	3.48 ± 0.24	−0.22 [−0.77, 0.33]	4.00 ± 0.21	0.31 [−0.18, 0.8]	0.094	0.91 [−0.07, 0.98]
Eosinophil/monocyte ratio	0.08 ± 0.00	0.08 ± 0.02	0.00 [−0.04, 0.04]	0.09 ± 0.02	0.01 [−0.02, 0.04]	0.448	0.40 [−0.49, 1.29]

CI; Confidence interval

### Chronic inflammatory cytokine responses to the training intervention

The resting levels of inflammatory cytokines were measured before and after the eight-week training intervention. Before analysis, the data underwent Log_10_ transformation and further normality testing after failing the Shapiro Wilk normality test. The ANCOVA showed no significant time × group interaction or time effect for any cytokine measured, and the effect sizes were unclear (*p* < 0.05; Table 3).

**Table 3:** Chronic inflammatory cytokine responses to resistance exercise in hypoxia; IL-1β, IL-4, IL-6, IL-8, IL-10 and TNFα in serum at pre-training and post-training. Values are adjusted means ± SE (*n* = 10). The p value reported is the time × group interaction

	Pre training	Normoxia		Hypoxia		Between group differences	
	Adjusted Mean ± SE	Post training Mean ± SE	Δ Change [95%C.I]	Post training Mean ± SE	Δ Change [95%C.I]	*P* value	Hedges G [95% CI]
IL-1β (pg/mL)	0.75 ± 0.00	0.83 ± 0.11	0.08 [−0.15, 0.31]	0.75 ± 0.11	0.00 [−0.24, 0.24]	0.508	−0.30 [−1.21, 0.61]
IL-4 (pg/mL)	35.9 ± 0.00	37.7 ± 3.82	1.80 [−6.29, 9.89]	35.4 ± 3.8	−0.55 [−8.64, 7.54]	0.500	−0.27 [−1.15, 0.61]
IL-6 (pg/mL)	1.49 ± 0.00	1.50 ± 0.14	−0.01 [−0.29, 0.31]	1.34 ± 0.15	−0.12 [−0.44, 0.19]	0.347	−0.44 [−1.38, 0.49]
IL-8 (pg/mL)	1.38 ± 0.00	1.65 ± 0.23	0.27 [−2.20, 0.76]	1.30 ± 0.23	−0.08 [−0.56, 0.41]	0.212	−0.64 [−1.65, 0.38]
IL-10 (pg/mL)	4.89 ± 0.00	5.28 ± 0.64	0.42 [−0.95, 1.78]	4.83 ± 0.68	−0.04 [−1.48, 1.41]	0.129	−0.70 [−1.63, 0.22]
TNF-α (pg/mL)	1.97 ± 0.00	2.11 ± 0.11	0.14 [−0.08, 0.37]	1.85 ± 0.10	−0.12 [−0.33, 0.01]	0.110	−0.11 [−1.06, 0.85]

IL: Interleukin. TNFα: tumor necrosis factor alpha. CI: Confidence interval

### Chronic hematological responses to the training intervention

There were no significant time × group interactions or main effects of time on RBCs, red cell distribution width, hemoglobin, hematocrit, mean corpuscular volume, platelets or mean platelet volume at rest (Table 4). However, there was a significant main time effect (*p* = 0.014) on the mean corpuscular haemoglobin concentration, where both groups increased in concentration after the training intervention, irrespective of hypoxia exposure (Table 4).

**Table 4:** Chronic hematological responses to resistance exercise in hypoxia; Red blood cells, hemoglobin, hematocrit and associated measures at pre- and post-training. Values are mean ± SE. *N* = 10. Adjusted values are reported from the ANCOVA output, using baseline and sex as covariates. The p value reported is the time × group interaction

	Pre training	Normoxia		Hypoxia		Between group differences	
	Adjusted Mean ± SE	Post training Mean ± SE	Δ Change [95% C.I]	Post training Mean ± SE	Δ Change [95% C.I]	P value	Hedges G [95% CI]
Red blood cells (×10^12^/L)	4.74 ± 0.00	4.73 ± 0.13	0.00 [−0.3, 0.3]	4.57 ± 0.11	−0.17 [−0.44, 0.10]	0.370	−0.47 [−1.41, 0.47]
Red cell distribution width (%)	13.93 ± 0.00	13.93 ± 0.15	−0.01 [−0.36, 0.34]	13.58 ± 0.13	−0.35 [−0.75, 0.05]	0.106	−0.88 [−1.85, 0.09]
Haemoglobin (g/L)	146.13 ± 0.00	147.36 ± 3.36	1.23 [−6.62, 9.08]	143.26 ± 2.98	−2.86 [−9.88, 4.16]	0.390	−0.45 [−1.39, 0.49]
Hematocrit (Fraction)	0.44 ± 0.00	0.44 ± 0.01	0.00 [−0.03, 0.03]	0.42 ± 0.01	−0.02 [−0.05, 0.01]	0.300	−0.57 [−1.52, 0.38]
Mean corpuscular volume (fL)	92.29 ± 0.00	92.29 ± 0.30	0.00 [−0.7, 0.7]	92.58 ± 0.27	0.29 [−0.34, 0.92]	0.498	0.37 [−0.57, 0.94]
Mean corpuscular haemoglobin (pg)	30.89 ± 0.00	31.13 ± 0.29	0.23 [−0.46, 0.92]	31.44 ± 0.26	0.55 [−0.07, 1.17]	0.437	0.43 [−0.51, 1.37]
Mean corpuscular haemoglobin concentration (g/L)	334.83 ± 0.00	337.43 ± 3.44	2.60 [−5.47, 10.67]	339.26 ± 3.08	4.42 [−2.8, 11.64]	0.698	0.20 [−0.73, 1.13]
Platelets (×10^9^/L)	239.33 ± 0.00	228.9 ± 9.68	−10.46 [−33.17, 12.25]	255.3 ± 8.66	15.97 [−4.34, 36.28]	0.087	1.04 [0.05, 2.03]
Mean platelet volume (fL)	9.15 ± 0.00	9.01 ± 0.11	−0.14 [0.4, 0.12]	9.26 ± 0.10	0.11 [0.11, 0.23]	0.135	0.81 [−0.16, 1.78]

CI: Confidence interval

### Acute leukocyte and cytokine responses after the training intervention

**Fig. 1:**
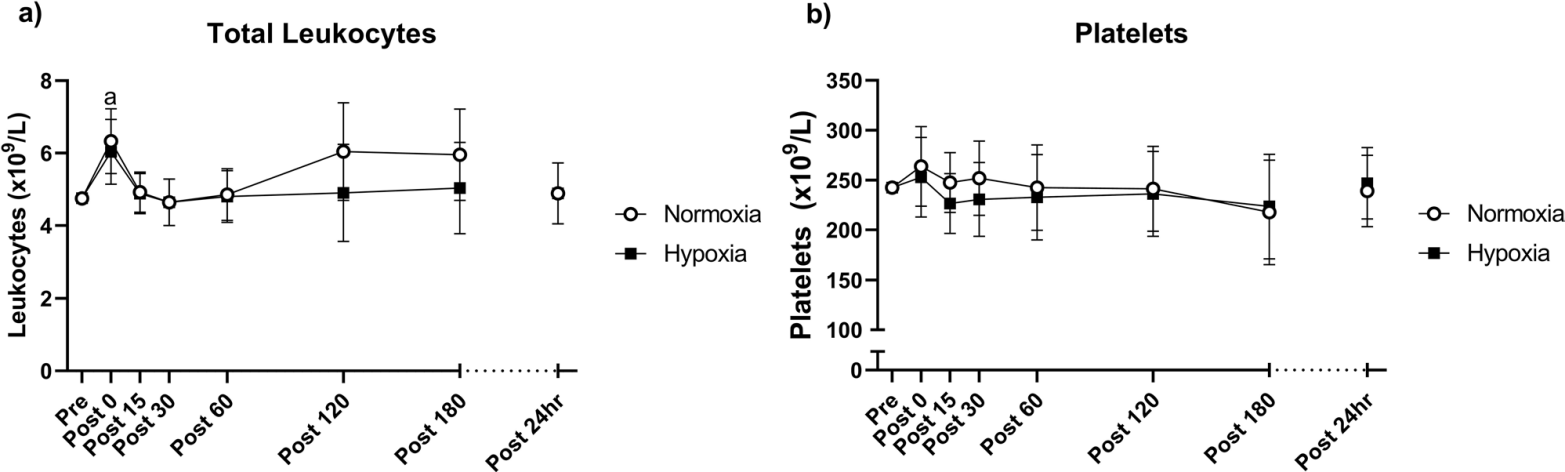
Total leukocytes (**a**) and platelets (**b**) before and in the 24 h following the final training session of the eight-week training intervention. Values are adjusted means ± SD (*n* = 10). Analyzed by a repeated measures ANCOVA. ^a^ represents a significant time effect (*p* < 0.05) compared to resting (pre) values

Leukocyte populations were measured at 0 min, and in the 24 h following the last training session of the eight-week intervention. Here, there were no statistically significant time x group interactions for total leukocytes or platelets (Fig. 1a-b). Irrespective of group allocation, total leukocytes were elevated at 0 min post-exercise (*p* < 0.001), although were not different to pre-exercise at any other time point (Fig. 1a).

**Fig. 2:**
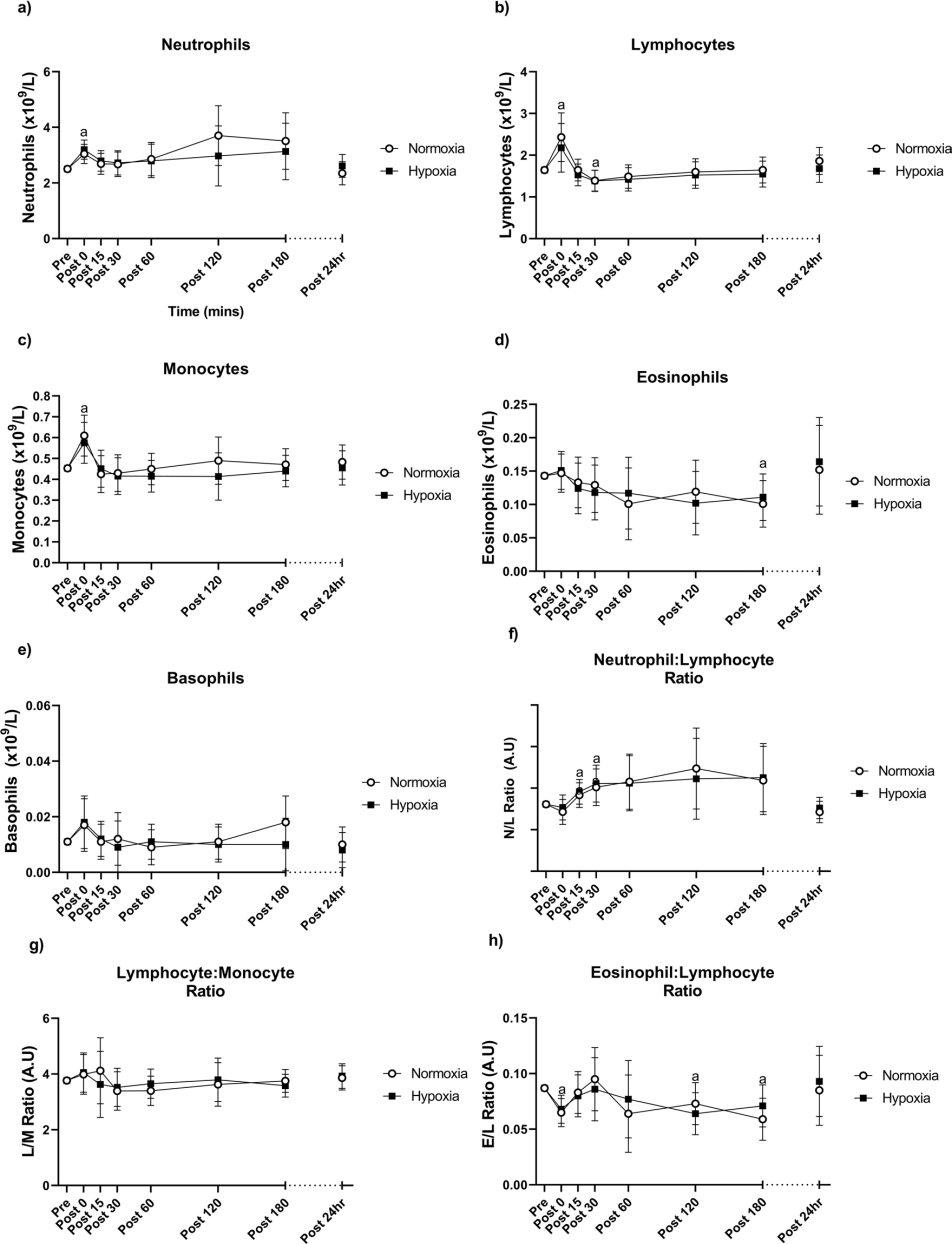
Neutrophils (**a**), lymphocytes (**b**), monocytes (**c**), eosinophils (**d**) and basophils (**e**), neutrophil-lymphocyte ratio (**f**), lymphocyte-monocyte ratio (**g**) and eosinophil-lymphocyte ratio before and in the 24 h following the final training session of the eight-week training intervention. Values are adjusted means ± SD (*n* = 10). Analyzed by a repeated measures ANCOVA. ^a^ represents a significant time effect (*p* < 0.05) compared to resting (pre) values

There were no significant time × group interactions present for neutrophils, lymphocytes, monocytes, eosinophils, basophils, neutrophil/lymphocyte ratio, lymphocyte/monocyte ratio or the eosinophil/lymphocyte ratio (Fig. 2a-h). There was a significant effect of time, where neutrophils and monocytes were significantly greater immediately after exercise (time = 0 min) compared to pre-exercise (*p* < 0.001), though were not different at any other time point. Similarly, total lymphocytes were elevated at 0 min post-exercise (*p* < 0.001), then dropped below pre-exercise levels at 30 min post-exercise (*p* = 0.004). Eosinophils dropped below pre-exercise levels at 180 min post-exercise (*p* = 0.005), though were not different to pre-exercise levels at 24 h post-exercise. The neutrophil/lymphocyte ratio rose above pre-exercise levels at 15 (*p* = 0.029) and 30 (*p* = 0.007) min post-exercise, whilst the eosinophil/lymphocyte ratio dropped below pre-exercise levels at 0 min (*p* < 0.001), 120 min (*p* = 0.015) and 180 min (*p* = 0.005) post-exercise. All other cell populations did not significantly change over the 24 h post-exercise (*p* < 0.05). At no point did hypoxia affect these concentrations.

**Fig. 3:**
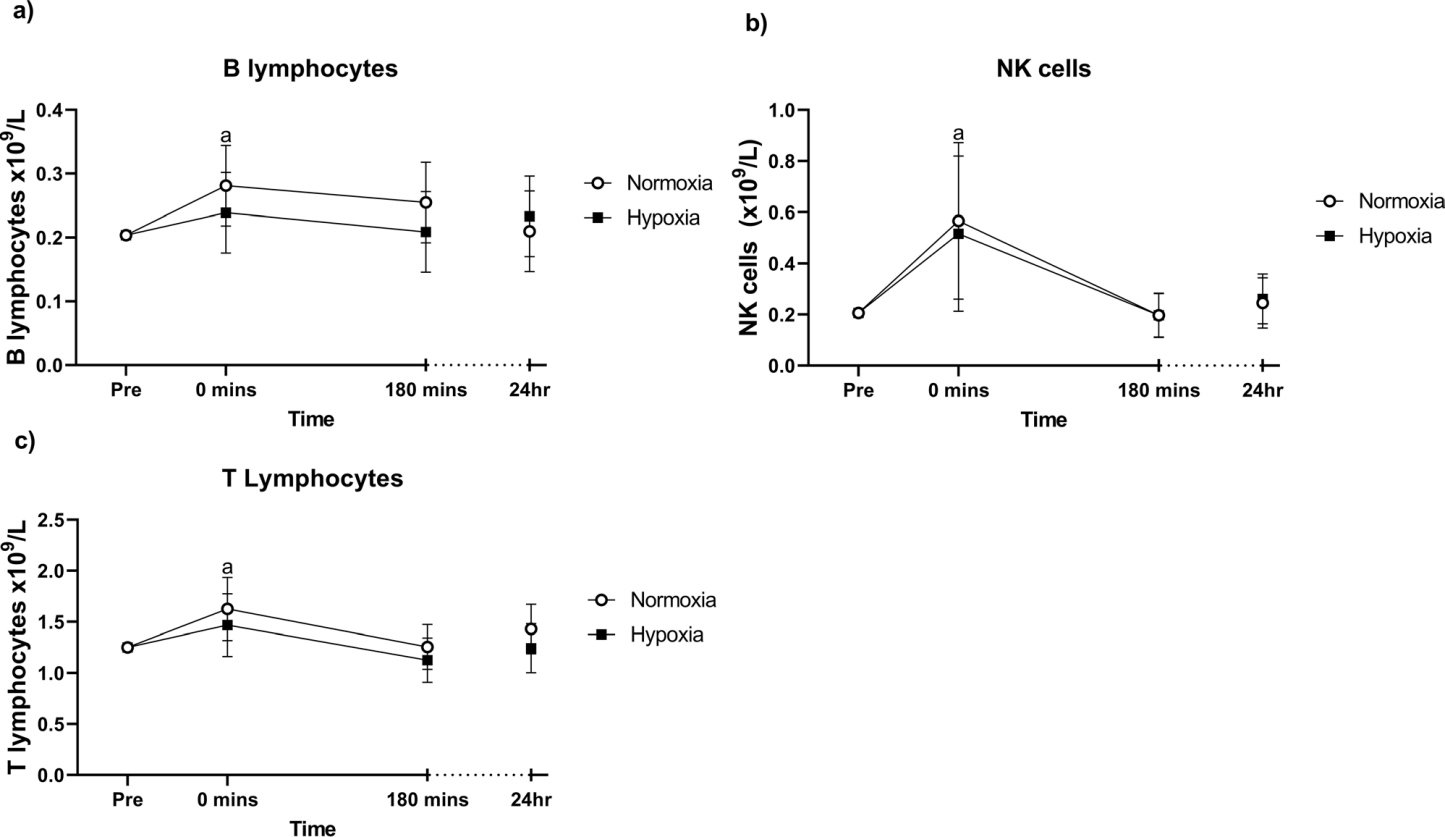
B lymphocytes (**a**), NK cells (**b**) and T lymphocytes (**c**) before and in the 24 h following the final training session of the eight-week training intervention. Values are adjusted means ± SD (*n* = 10). Analyzed by a repeated measures ANCOVA, using sex and baseline as covariates. ^a^ represents a significant time effect (*p* < 0.05) compared to resting (pre) values

Flow cytometric analysis of lymphocyte subpopulations was performed at three selected time points; 0 min, 180 min and 24 h. There were no significant time × group interactions for B lymphocytes, NK cells or T lymphocytes (Fig. 3a-c). Irrespective of group allocation, at 0 min post-exercise, B lymphocytes (*p* = 0.002), NK cells (*p* = 0.001) and T lymphocytes (*p* = 0.002) were significantly higher than pre-exercise levels, although were not different at any other time point. At no point did hypoxia affect these concentrations.

**Fig. 4:**
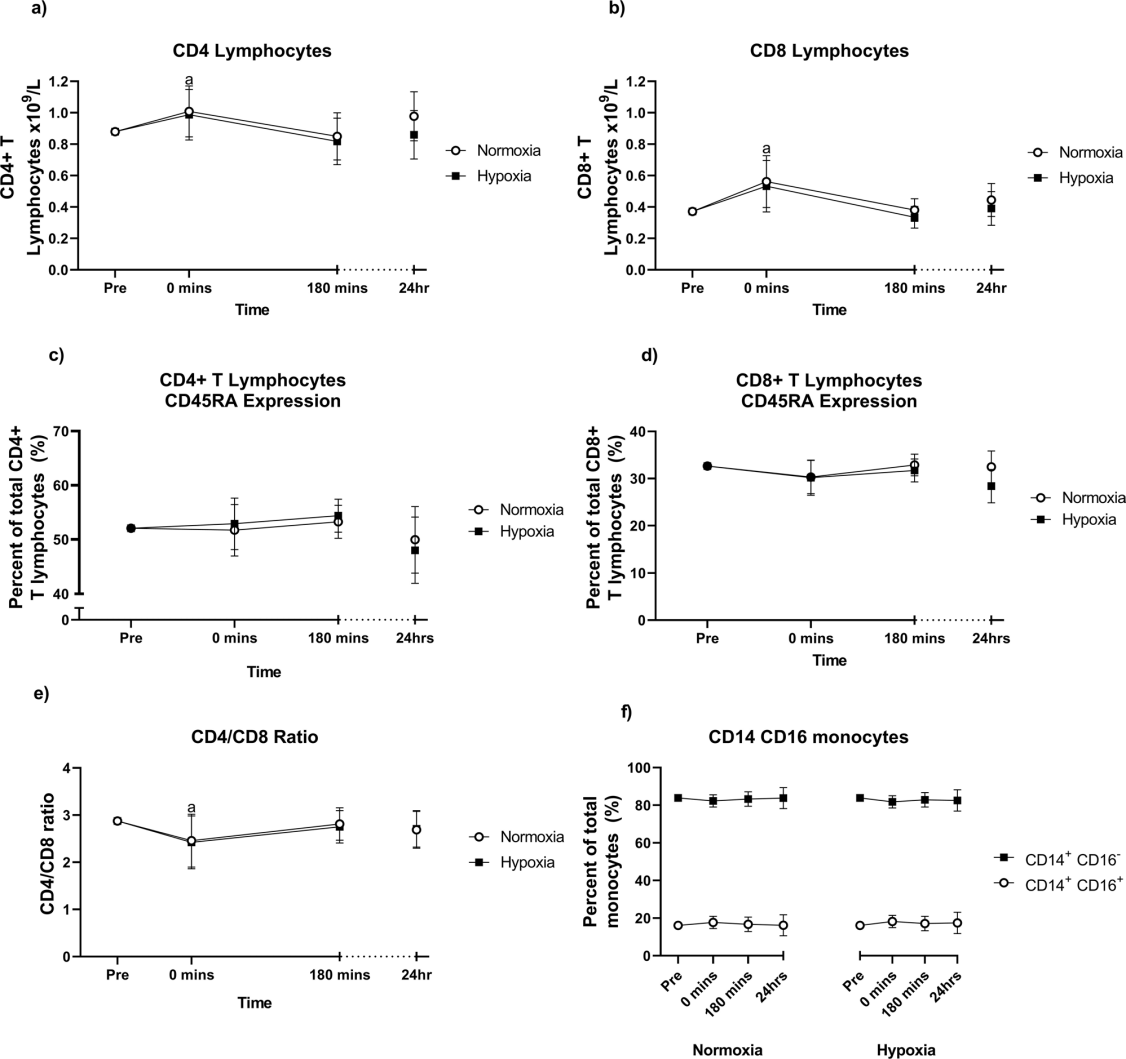
CD4^+^ T helper cells (**a**), CD8^+^ T cytotoxic cells, the subsets of CD45RA^+^ and CD45RA^−^ T cells (**c**, **d**), the CD4/CD8 ratio (**e**) and the subsets of CD14^+^ CD16^−^ (classical) and CD14^+^ CD16^+^ (non-classical/intermediate) monocytes (**f**) before and in the 24 h following the final training session of the eight-week training intervention. Values are adjusted means ± SD (*n* = 10). Analyzed by ANCOVA. ^a^ represents a significant time effect (*p* < 0.05) compared to resting (pre) values

Analysis of T cell and monocyte subsets showed no significant time × group interaction for CD4^+^ T helper cells, CD8^+^ cytotoxic T cells, CD4/8 ratio, CD14^+^ CD16^+^ monocytes, CD14^+^ CD16^−^ monocytes or the expression of CD45RA on CD4^+^ T cells and CD8^+^ T cells (Fig. 4a-f). At 0 min post-exercise, CD4^+^ T cells and CD8^+^ T cells were significantly higher than pre-exercise levels, although were not different at any other time point. The CD4/CD8 ratio was significantly lower 0 min post-exercise, compared to pre-exercise levels (*p* = 0.021). At no point did hypoxia affect these concentrations.

**Fig. 5:**
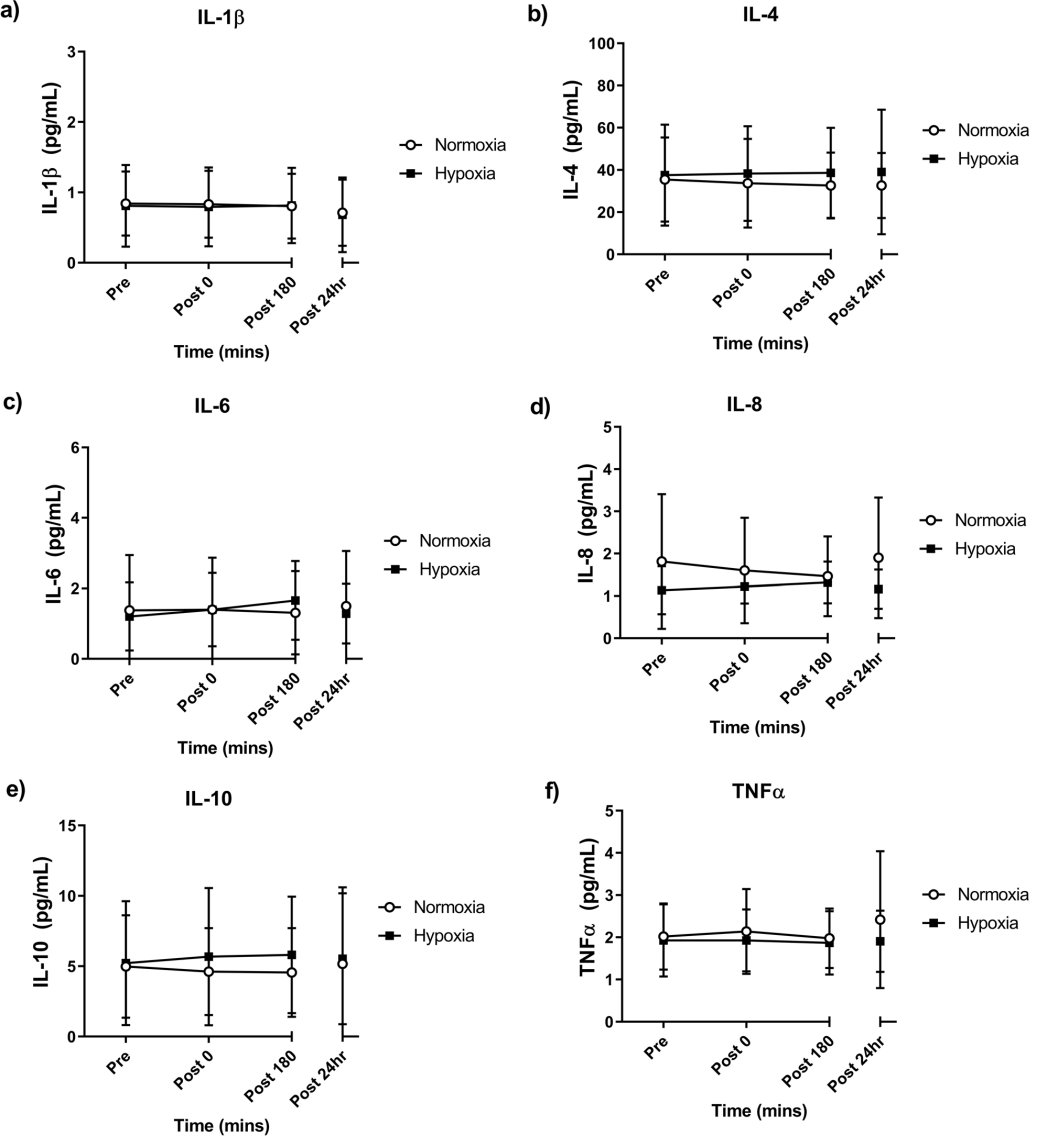
Acute Interleukin-1 beta (IL-1β; **a**) Interleukin-4 (IL-4; **b**), Interleukin-6 (IL-6; **c**), Interleukin-8 (IL8; **d**), Interleukin-10 (IL-10; **e**) and tumor necrosis factor alpha (TNF-α; **f**) pre- and post-exercise. Values are mean ± SD (*n* = 10). Analyzed using an ANCOVA

There were no significant time × group interactions present for any inflammatory cytokine measured in the 24 h following the last training session; IL-1β (*p* = 0.844), IL-4 (*p* = 0.389), IL-6 (*p* = 0.1), IL-8 (*p* = 0.417), IL-10 (*p* = 0.185) and TNFα (*p* = 0.358; Fig. 5a-f). There were no effects of time over the 24 h measurement period for any cytokine measured (*p* < 0.05).

## Discussion

Older adults typically experience a decline in the number and function of immunological parameters (1) that increases their risk of illness (2) and infection (3). Acute resistance exercise [21] and passive exposure to hypoxia [28] can augment immunological parameters in the circulation of older adults. Therefore, this study characterized the chronic leukocyte and cytokine responses of older adults to eight-weeks of resistance training in hypoxia. We hypothesized that training in hypoxia for eight weeks would cause a greater increase in the resting concentration of leukocytes and anti-inflammatory cytokines, and a greater decrease in pro-inflammatory cytokines compared to the same training in normoxia. We also hypothesized that after the eight-week training intervention, the hypoxic group would show greater acute leukocyte and cytokine responses to the resistance training protocol, compared to the same exercise in normoxia. This study showed that after eight weeks of resistance training, the concentration of resting T cells, B cells, NK cells and eosinophils was higher in the hypoxic group compared to the normoxic group, however the inflammatory cytokines were unchanged. Contrary to our hypothesis, hypoxia did not affect the leukocyte or cytokine responses in the 24 h following the final training session of the intervention.

After the eight-week training intervention the resting concentration of leukocytes was 20.2% higher in hypoxia when compared to normoxia. This novel finding was not surprising, given the clear links between hypoxia exposure and leukocyte recruitment into the systemic circulation [28]. Lymphocytes were the main contributor to the higher total leukocyte concentration after training in hypoxia, with NK cells, T and B lymphocytes 36.0%, 22.6% and 28.0% greater than normoxia. Future studies should investigate if these changes could confer an increased capacity to eliminate pathogens and their capacity for cellular signaling with other immune cells and inflammatory cytokines. Further lymphocyte function and senescence assays are required to fully understand the lymphocyte responses to hypoxic resistance training. The mechanism causing the higher lymphocyte concentrations in hypoxia is currently unclear, although hypoxia is generally known to increase lymphocyte proliferation, survival and glycolytic function [36].

Flow cytometric characterization of the T lymphocyte subpopulations showed that the resting CD4:CD8 T cell ratio was unresponsive to either hypoxia or the resistance training intervention. The CD4:CD8 ratio was above 2.0 in both groups before the training intervention and remained above 2.0 after the intervention. A CD4:CD8 ratio below 1.0 forms part of the criteria that predicts all-cause mortality in adults aged 80–99 years [16] and therefore the older adult population in this study were classified as ‘healthy’. However, the number of CD8^+^ T cytotoxic cells after the training intervention was greater in hypoxia compared to normoxia. On one hand, this change could signify an increased capacity of older adults to mount a CD8^+^ cytotoxic T cell response to invading pathogens. On the other hand, the increased CD8^+^ T cells may be unfavorable as it drives the CD4:CD8 ratio downwards. Further research is required to explore the functional CD8^+^ T cell responses to resistance training in hypoxia, with an emphasis on the cytolytic capacity of these cells.

CD4^+^ and CD8^+^ T cells were stained with CD45RA as a basic marker of cell senescence, where naïve cells are typically CD45RA^+^ and senescent cells are typically CD45RA^−^ [34]. Although more T cell markers are required to accurately characterize cell senescence, the analysis showed that the expression of CD45RA on CD4^+^ and CD8^+^ T cells at rest was unaffected by hypoxia. Although hypoxia did not affect CD4^+^ or CD8^+^ T cell senescence in this study, four weeks of hypoxic (15% O_2_) endurance training in young males can significantly reduce the proportion of senescent T cells (KLRG1^+^) in the circulation, using KLRG1 as a cell marker [37]. Interestingly, independent of group allocation the eight-week resistance training intervention reduced the resting proportion of CD45RA^−^ CD8^+^ T cells, suggesting that there may have been a reduction in senescent CD8^+^ T cells. This response is likely beneficial as senescent CD8^+^ T cells have a lower cytolytic and proliferative capacity than naïve T cells [15]. Although the literature is scarce in this area, exercise training is thought to reduce senescent T cells through their repeated recruitment into peripheral tissues, where they are more likely to undergo apoptosis [15]. Given that the T cell pool is likely capped, apoptosis of senescent T cells may create space for naïve T cell expansion and thus reduce T cell senescence [38]. More research is required to explore these effects with a greater number of senescence markers and participants.

The only other subpopulation of resting leukocytes that were chronically affected by hypoxia were eosinophils. Eosinophils were 36.4% higher in hypoxia after the training intervention when compared to normoxia, though the concentrations remained within normal ranges for the Australian population [39]. Eosinophils are vital for the elimination of pathogens and play a key role in allergic reactions [40]. Therefore, further research is required to examine if the subtle change in resting eosinophils with hypoxic resistance training confers a functional benefit to pathogen removal or the response to allergens.

The combination of resistance training and normobaric hypoxia did not increase resting red blood cell concentration, haemoglobin or hematocrit. Although normoxic resistance training does not consistently increase hematological parameters in older adults [23], five days of intermittent hypoxia exposure (SpO_2_ ∼85%) in healthy older adults increased red blood cell and hemoglobin concentrations by 8% and 15% respectively, when compared to normoxic controls [41]. Erythropoiesis is a classic response to hypoxia exposure [42], however the severity, duration and frequency of hypoxic exposure in this study was likely insufficient to elicit erythropoiesis. Hemoglobin mass is estimated to increase by ∼1.1% per 100 h spent at an altitude of > 2,100 m [43]. Increasing the severity of the hypoxic dose and the frequency of training would likely promote erythropoiesis, although this would increase the physiological risk and reduce the feasibility of such programs outside the laboratory setting.

Analysis of resting inflammatory cytokines showed no significant changes to the resting levels of IL-1β, IL-4, IL-6, IL-8, IL-10 or TNFα following the training intervention in hypoxia or normoxia. None of the cytokine populations exceeded the normal reference ranges for healthy adults at baseline [44], possibly explaining the null findings. Interestingly, treatment with intermittent hypoxia (12–15% O_2_) for eight weeks alone can increase the circulating levels of the anti-inflammatory cytokine IL-10 in young males [45]. Another study demonstrated a reduction in systemic TNFα and IL-4 after exposing healthy young males to 10% O_2_ intermittently for 14 days [46]. The only study combining resistance training and hypoxia used a mild hypoxic dose (16.1% O_2_) in older adults for 24 weeks, and found no changes to systemic IL-6, IL-8 or IL-10 [47]. Kiers et al. suggest that hypoxia attenuates pro-inflammatory cytokines and increases anti-inflammatory cytokines when faced with an inflammatory challenge (via an adenosine 2B receptor-dependent post-transcriptional mechanism), however we did not observe this phenomenon in our study [48]. In summary, it appears that combining moderate hypoxia exposure with resistance training does not improve resting levels of inflammatory cytokines in healthy older adults. A higher frequency of training or a longer training period may have been more likely to alter cytokine concentrations.

Additional blood samples were drawn following the last training session of the eight-week training intervention to capture any effects of hypoxia on the acute leukocyte response to resistance exercise. The time points measured represent the most thorough characterization of the leukocyte response following a bout of resistance training in older adults in the literature, where blood was sampled frequently over the 3 h post-exercise and again at 24 h post-exercise. There were no significant impacts of hypoxia on the acute leukocyte or platelet responses to the last training session of the intervention. This result was somewhat surprising given that in our previous study, exposure to a single session of hypoxic resistance exercise in untrained older adults increased the number of circulating lymphocytes over 24 h post-exercise compared to the same training in normoxia [31]. Interestingly, we also previously showed a blunted growth hormone response to hypoxic resistance exercise in older adults after an eight-week training period, compared to the same resistance exercise in normoxia [27]. Given that growth hormone stimulates lymphocyte proliferation [49], this result could partly explain the null findings. Further research is required to determine the physiological cause for this response with a greater sample size.

Analysis of the three lymphocyte subsets (B, T and NK cells) also showed no significant effects of hypoxia on the acute response to the last training session. Further characterization of monocyte subsets, the T lymphocyte subsets and their expression of CD45RA showed no significant effects of hypoxia on the acute response to exercise. These findings contrast our previous research that untrained older adults show a greater lymphocyte response to a single bout of resistance exercise in hypoxia compared to normoxia [31]. Although hypoxia is a known stimulator of leukocyte populations [28], it appears that the immune response to acute hypoxic resistance exercise in untrained older adults is short lived and is no longer present after a period of training. Finally, the acute cytokine response to the final training session was not impacted by hypoxia. These null responses were consistent with our previous findings that older adults do not experience a greater acute cytokine response to a single bout of resistance exercise in moderate hypoxia, compared to normoxia [31].

There were several limitations to our study, including the relatively small sample size and the inherent variability in the quantification of inflammatory cytokines. Future studies may look to expand these methods to a larger cohort of participants and ensure that the study controls for factors such as time of day, hydration status and illness.

## Conclusions

Hypoxia caused higher resting lymphocyte and eosinophil concentrations in the systemic circulation after 8 weeks of resistance training, when compared to normoxia. Our findings suggest that chronic resistance training in hypoxia is not detrimental to immune cell number in older adults, and may have an additional beneficial effect on the immunological status of older adults. More research is needed to determine the causes and implications of these responses.

### Electronic supplementary material

Below is the link to the electronic supplementary material.

Supplementary Material 1

## Data Availability

The source data are available to verified researchers upon request by contacting the corresponding author.

## Abbreviations

ANCOVA: analysis of covariance
BMI: body mass index
CD: Cluster of differentiation
CRP: Cre-reactive protein
Interleukin: IL
MCH: mean corpuscular hemoglobin
MCHC: MCH concentration
MCV: mean corpuscular volume
NK: Natural killer
MPV: platelets and mean platelet volume
RDW: red cell distribution width
RM: repetition maximum
O_2_: oxygen
SpO_2_: peripheral oxygen saturation
TNFα: Tumor necrosis factor alpha
